# Identification and evolutionary characterization of salt-responsive transcription factors in the succulent halophyte *Suaeda fruticosa*

**DOI:** 10.1371/journal.pone.0222940

**Published:** 2019-09-23

**Authors:** Joann Diray-Arce, Alisa Knowles, Anton Suvorov, Jacob O’Brien, Collin Hansen, Seth M. Bybee, Bilquees Gul, M. Ajmal Khan, Brent L. Nielsen

**Affiliations:** 1 Department of Microbiology and Molecular Biology, Brigham Young University, Provo, Utah, United States of America; 2 Department of Biology, Brigham Young University, Provo, Utah, United States of America; 3 Institute of Sustainable Halophyte Utilization, University of Karachi, Karachi, Pakistan; University of Western Sydney, AUSTRALIA

## Abstract

Transcription factors are key regulatory elements that affect gene expression in response to specific signals, including environmental stresses such as salinity. Halophytes are specialized plants that have the ability to complete their life cycle in saline environments. In this study we have identified and characterized the evolutionary relationships of putative transcription factors (TF) in an obligate succulent halophyte, *Suaeda fruticosa*, that are involved in conferring salt tolerance. Using RNA-seq data we have analyzed the expression patterns of certain TF families, predicted protein-protein interactions, and analyzed evolutionary trajectories to elucidate their possible roles in salt tolerance. We have detected the top differentially expressed (DE) transcription factor families (MYB, CAMTA, MADS-box and bZIP) that show the most pronounced response to salinity. The majority of DE genes in the four aforementioned TF families cluster together on TF phylogenetic trees, which suggests common evolutionary origins and trajectories. This research represents the first comprehensive TF study of a leaf succulent halophyte including their evolutionary relationships with TFs in other halophyte and salt-senstive plants. These findings provide a foundation for understanding the function of salt-responsive transcription factors in salt tolerance and associated gene regulation in plants.

## Introduction

Salinity causes significant losses in agricultural production due to the limited capacity of crops to regulate homeostasis [1]. Halophytes are specialized plants that have adapted to tolerate high salt concentrations through complex mechanisms of gene expression and protein pathway adaptation [2]. In adverse environments, halophytes utilize a variety of physiological and metabolic responses to regulate stress-responsive genes and synthesize functional proteins through a complex signal transduction network to confer salinity tolerance [1]. Moreover, functional salt tolerance requires integrated adaptations from cellular systems to the whole plant to satisfy energy needs [3].

Transcription factors (TF) are proteins that bind to specific DNA sequences to control the rate of transcription of target genes and are essential regulators for gene expression in response to environmental signals [4]. TFs are necessary for controlling cellular processes including the regulation of intercellular mechanisms, cell cycle, growth and reproduction, and stress responses, making TF characterization extremely valuable [5, 6]. Some TFs alter expression of genes to enhance tolerance to harsh environments [7], and many of these are conserved in plants. Despite the wealth of genomic and transcriptomic information on glycophytes and halophytes, there are still many unknown aspects of plant strategies for survival, tolerance and productivity at specific salt concentrations.

New high-throughput technologies allow for the generation of data that address questions of temporal and spatial responses to a variety of stresses and enable more structured gene expression prediction and plant mechanism characterization [8]. Transcriptomic studies have been used to analyze stress-related conditions in crops; however, meta-analysis research on specialized plants including halophytes is very limited [9]. Although there have been studies of differentially expressed genes in relation to salt tolerance, studies on plant signaling components and key regulators of salt responses, and the evolutionary relationships of these TFs across plant families, are lacking. Therefore, integration and identification of TFs in adaptive signaling networks are key factors for understanding the adaptations of plants to environmental stress [6].

*Suaeda fruticosa* Forssk is a perennial leaf succulent halophyte that sequesters NaCl into its vacuoles. Optimal growth of this species occurs at 300 mM NaCl, where plants increase the concentration of leaf Na^+^ and Ca^2+^, creating conditions for enhanced water absorption, while other physiological parameters function normally [10]. Sodium ion buildup begins rapidly at 600 mM NaCl, increasing in ion toxicity leading to a compromised antioxidant system and substantial growth reduction [10]. We utilized RNA-sequencing to assemble the transcriptome and identify differentially expressed genes for this obligate halophyte [11]. In the present study, the *S*. *fruticosa* transcriptome data were analyzed to extract TF sequence information in order to identify family groups and characterize gene expression patterns in shoot and root tissue under long-term low (0 mM NaCl) or optimum (300 mM NaCl) salinity treatment. We have validated these findings using qRTPCR, including analysis of data from high (900 mM NaCl) salinity treatment. Hidden Markov model-based domain searches and BLAST-based protein homology searches were used to predict TFs [12]. We reconstructed transcription factor family trees found in PlantTFDBv3.0 to determine the evolutionary relationships of differentially expressed TFs versus non-differentially expressed TFs in *S*. *fruticosa*. We focused on TF families with the highest numbers of differentially expressed genes (MYB, CAMTA, MADS-box and bZIP) to determine their characteristics and evolutionary relationships.

## Materials and methods

### Transcription factor (TF) mining and differential gene expression (DEG) analysis

A description of plant samples processed for RNA-Seq and methods for bioinformatics analysis including *de novo* assembly and differential expression analysis was described earlier [11]. RNA-Seq Illumina sequences are available at the NCBI Sequence Read Archive under *Suaeda fruticosa* accession SRX973396. Transcriptome sequence information is deposited in the Transcriptome Shotgun Assembly Sequence Database: BioProject ID: PRJNA279962 and PRJNA279890. The supplementary information files will be publicly available at Dryad upon acceptance. Differentially expressed (DE) genes and the entire assembled transcriptome were translated using Transdecoder software and the protein sequences clustered using CD-HIT [13].

**Fig 1:**
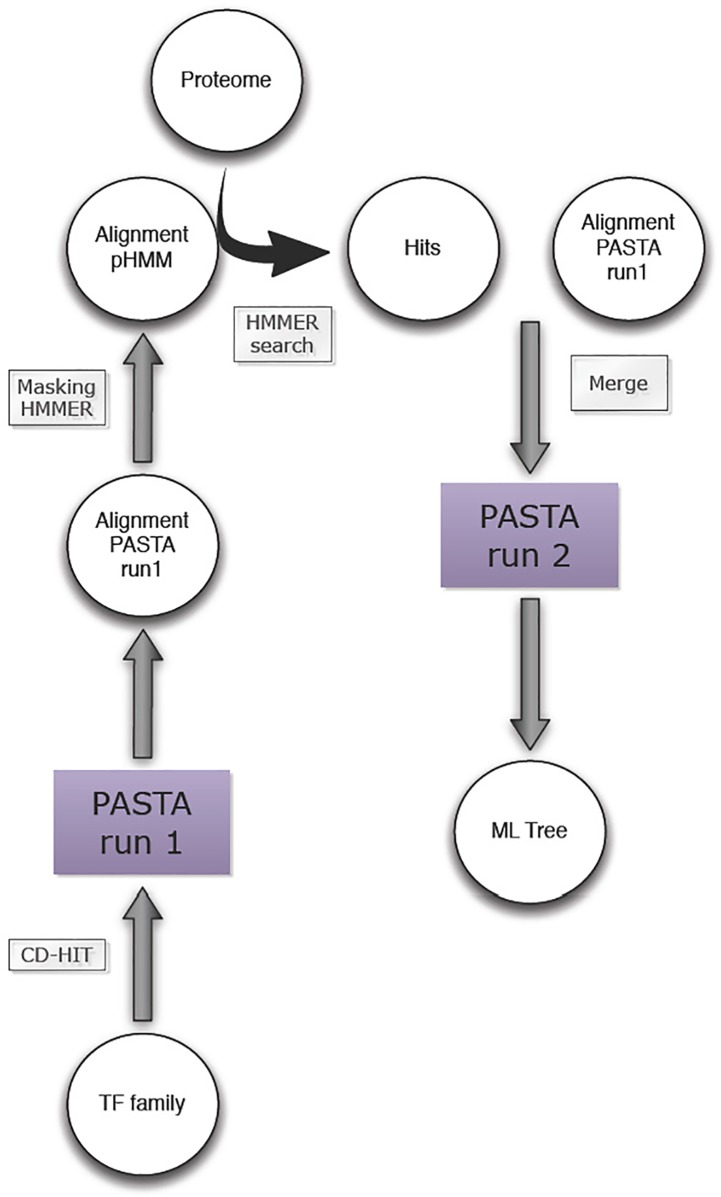
Diagram of the tree inference workflow used. The maximum likelihood gene tree was constructed using a co-estimation algorithm in two iterations of PASTA. Results from a profile hidden Markov search in HMMER (E-value cutoff of 10^−10^) were then combined with the original TF family sequences and analyzed with another iteration of co-estimation.

Transcription factors were identified and used to search against the Plant Transcription Factor Database 3.0. HMM profiles of the 57 families were obtained and used to search against the *S*. *fruticosa* proteome using profile hidden Markov search in HMMER with an E-value cutoff of 10^−10^. Codes for TF prediction, DE TF identification and phylogenetic tree construction are available (S5 Fig). To identify potential TFs in the transcriptome and classify to which family each belongs to, we utilized HMM-based TF domain identification and protein homology search on the available transcriptome sequences of *S*. *fruticosa* (Fig 1).

Analysis of differential expression between treatments of 0 mM and 300 mM NaCl from *S*. *fruticosa* was performed using the EdgeR package from R [14]. We used the generalized linear models for data analysis for different salt concentrations of treatment and biological replicates. This differentiates the number of expressed transcripts across experimental conditions. We then searched and identified TF from the differentially expressed list using a profile hidden Markov search in HMMER [15] using an E-value of 10^−10^ against the database from PlantTFDBv3.0. These TF were annotated based on gene ontology, and their functional domains and structures using BLAST2GO against NCBI non-redundant (nr) and SwissProt protein databases with a similar E-value cutoff of 10^−10^. Enrichment analysis for specific gene ontology for biological process, molecular function and cellular components were determined using default parameters. Functional interactions between DE TFs were performed using STRING software version 10. STRING is a widely used database and web interface to explore protein-protein interactions, including physical and functional interactions [16].

We performed protein sequence comparison of *S*. *fruticosa* TFs to known TFs of other green plant species using NCBI BLAST (Table 1). *S*. *fruticosa* protein sequences were generated in Fasta, and multiple sequence alignment was performed using EMBL-EBI Clustal Omega version using default parameters with input order.

**Table 1:** Identification of TFs in *S*. *fruticosa* by comparison with known TFs of other green plant species using NCBI BLAST.

TF	Suaeda fructicosa Locus ID	Comparison	Accession Number	Query Cover	E Value	Identity
**CAMTA**						
CAMTA10	Locus_5187_Transcript_1/9984|m.39710	Calmodulin-binding transcription activator 2-like protein [Spinacia oleracea]	XP_021860116	95%	4e^-72	43.42%
CAMTA11	Locus_5187_Transcript_2/9990|m.39711	Calmodulin-binding transcription activator 2-like protein [Spinacia oleracea]	XP_021860116	98%	1e^-110	54.03%
CAMTA12	Locus_5187_Transcript_9/10010|m.39712	Calmodulin-binding transcription activator 2-like protein [Spinacia oleracea]	XP_021860116	94%	5e^-105	56.11%
CAMTA10	Locus_5187_Transcript_1/9984|m.39710	Calmodulin-binding transcription activator 3-like isoform X2 [Chenopodium quinoa]	XP_021752820	95%	1e^-173	68.32%
CAMTA11	Locus_5187_Transcript_2/9990|m.39711	Calmodulin-binding transcription activator 3-like isoform X2 [Chenopodium quinoa]	XP_021752820	94%	8e^-161	74.52%
CAMTA12	Locus_5187_Transcript_9/10010|m.39712	Calmodulin-binding transcription activator 3-like isoform X2 [Chenopodium quinoa]	XP_021752820	95%	3e^-154	75.17%
**BZIP**						
BZIP57	Locus_50829_Transcript_4/59749|m.38957	bZIP transcription factor 16-like isoform X2 [Spinacia oleracea]	XP_021851865	98%	4e^-175	84.10%
BZIP59	Locus_50829_Transcript_7/59751|m.38959	bZIP transcription factor 16-like isoform X2 [Spinacia oleracea]	XP_021851865	95%	9e^-129	88.27%
BZIP60	Locus_50829_Transcript_8/59752|m.38960	bZIP transcription factor 16-like isoform X2 [Spinacia oleracea]	XP_021851865	99%	0	85.75%
BZIP57	Locus_50829_Transcript_4/59749|m.38957	bZIP transcription factor 16-like [Chenopodium quinoa]	XP_021768278	98%	3e^-167	83.39%
BZIP59	Locus_50829_Transcript_7/59751|m.38959	bZIP transcription factor 16-like [Chenopodium quinoa]	XP_021768278	95%	7e^-117	87.24%
BZIP60	Locus_50829_Transcript_8/59752|m.38960	bZIP transcription factor 16-like [Chenopodium quinoa]	XP_021768278	99%	0	85.75%
**MYB**						
MYB07	Locus_37251_Transcript_2/44957|m.27707	transcription factor MYB13-like [Spinacia oleracea]	XP_021854979	95%	5e^-62	55.28%
MYB37	Locus_17372_Transcript_12/25781|m.12237	transcription factor MYB124 isoform X1 [Spinacia oleracea]	XP_021844608	99%	1e^-96	83.43%
MYB72	Locus_36812_Transcript_1/44096|m.27172	Protein LHY isoform X3 [Spinacia oleracea]	XP_021845986	99%	0	77.28%
MYB07	Locus_37251_Transcript_2/44957|m.27707	transcription factor MYB14-like [Chenopodium quinoa]	XP_021770182	95%	9e^-91	63.82%
MYB37	Locus_17372_Transcript_12/25781|m.12237	transcription factor MYB124-like [Chenopodium quinoa]	XP_021746303	99%	0	81.79%
MYB72	Locus_36812_Transcript_1/44096|m.27172	Protein LHY-like isoform X1 [Chenopodium quinoa]	XP_021748820	99%	0	82.16%
**M-TYPE**						
M-Type26	Locus_82944_Transcript_1/77047|m.54026	MADS-box protein AGL24-like [Spinacia oleracea]	XP_021860771	91%	1e^-66	65.97%
M-Type28	Locus_82944_Transcript_4/77050|m.54028	MADS-box protein AGL24-like [Spinacia oleracea]	XP_021860771	83%	6e^-74	65.43%
M-Type29	Locus_82944_Transcript_6/77052|m.54029	MADS-box protein AGL24-like [Spinacia oleracea]	XP_021860771	98%	1e^-87	61.03%
M-Type26	Locus_82944_Transcript_1/77047|m.54026	MADS-box protein AGL24-like [Chenopodium quinoa]	XP_021773608	91%	4e^-61	63.19%
M-Type28	Locus_82944_Transcript_4/77050|m.54028	MADS-box protein AGL24-like [Chenopodium quinoa]	XP_021773608	83%	2e^-67	62.35%
M-Type29	Locus_82944_Transcript_6/77052|m.54029	MADS-box protein AGL24-like [Chenopodium quinoa]	XP_021773608	98%	4e^-88	60.09%

### Molecular and evolutionary analysis of gene structure and motif composition of selected TF families

In order to generate multiple sequence alignment of an entire TF family and construct a corresponding Maximum-Likelihood (ML) gene tree we used an alignment-tree co-estimation algorithm implemented in PASTA [17]. PASTA has been shown to produce accurate alignments and generate trees on large datasets. First, we ran PASTA for two iterations to generate TF family alignments and masked sites with <5% data. Second, we used that masked alignment to extract homologous genes from the *S*. *fruticosa* transcriptome using profile hidden Markov search in HMMER [15] with the E-value cutoff of 10^−10^. These gene hits were then combined with the original TF family sequences and the alignment and tree was co-estimated again in PASTA (Fig 1). Constructed trees from all plant TF families are uploaded and can be viewed using FigTree from this source: (will be deposited in Dryad repository upon acceptance).

### Validation of differentially expressed transcription factors

*Suaeda fruticosa* seedlings were grown at Brigham Young University, Provo, Utah, USA according to the optimized protocol [10] under long term salinity treatment. After 8 weeks of growth, NaCl (0, 300 and 900 mM) was gradually introduced at the rate of 150 mM NaCl after 48 h intervals to avoid osmotic shock in such a way that all final salinity concentrations were achieved on the same day [10]. Plant samples of three biological replicates from roots and shoots were treated at low (0 mM NaCl) and optimal (300 mM NaCl) salt conditions and used for transcriptome sequencing. For qRTPCR analysis RNA was isolated from 0 mM, 300 mM, and 900 mM NaCl (high inhibitory) grown plants.

Transcription factors identified were selected for validation of differential expression using qRTPCR. For each qRTPCR reaction, 1 μg of RNA of 0 mM, 300 mM and 900 mM NaCl treated samples were reverse transcribed into cDNA using oligodT primers and Superscript IV (Life Technologies), and the cDNA libraries produced were used for qRTPCR as described [18]. The 900 mM samples were included as this high concentration is very inhibitory to plant growth, providing another comparison point. Primer sequences are available as supplementary information (S1 File). We ran second strand synthesis using an ABI Plus One thermocycler with annealing temperature of 58°C. To assess validation for each gene, qRTPCR data were analyzed based on ΔΔCT and 2^-ΔΔCT^ method. The ΔCT value of each gene was calculated by subtracting the CT value of the endogenous control from the CT value of the target gene.

We selected the alpha tubulin gene as an endogenous control. Primers were designed from the top DE TF from *S*. *fruticosa* transcriptome sequences and optimized for RTPCR. We chose to sample 3 gene targets per family. Expression analysis using ΔΔCT, 2^-ΔΔCT^ and standard error of the mean were calculated using the data analysis package in Microsoft Excel. Data were plotted as mean fold change (2^-ΔΔCT^). Statistically significant differences (p < 0.05) were determined using a one-tailed two-sample t-test assuming equal variances for comparison of the fold change values between biological replicates using GraphPad Prism software.

## Results

### Molecular characterization of abundant transcription factor families

**Fig 2:**
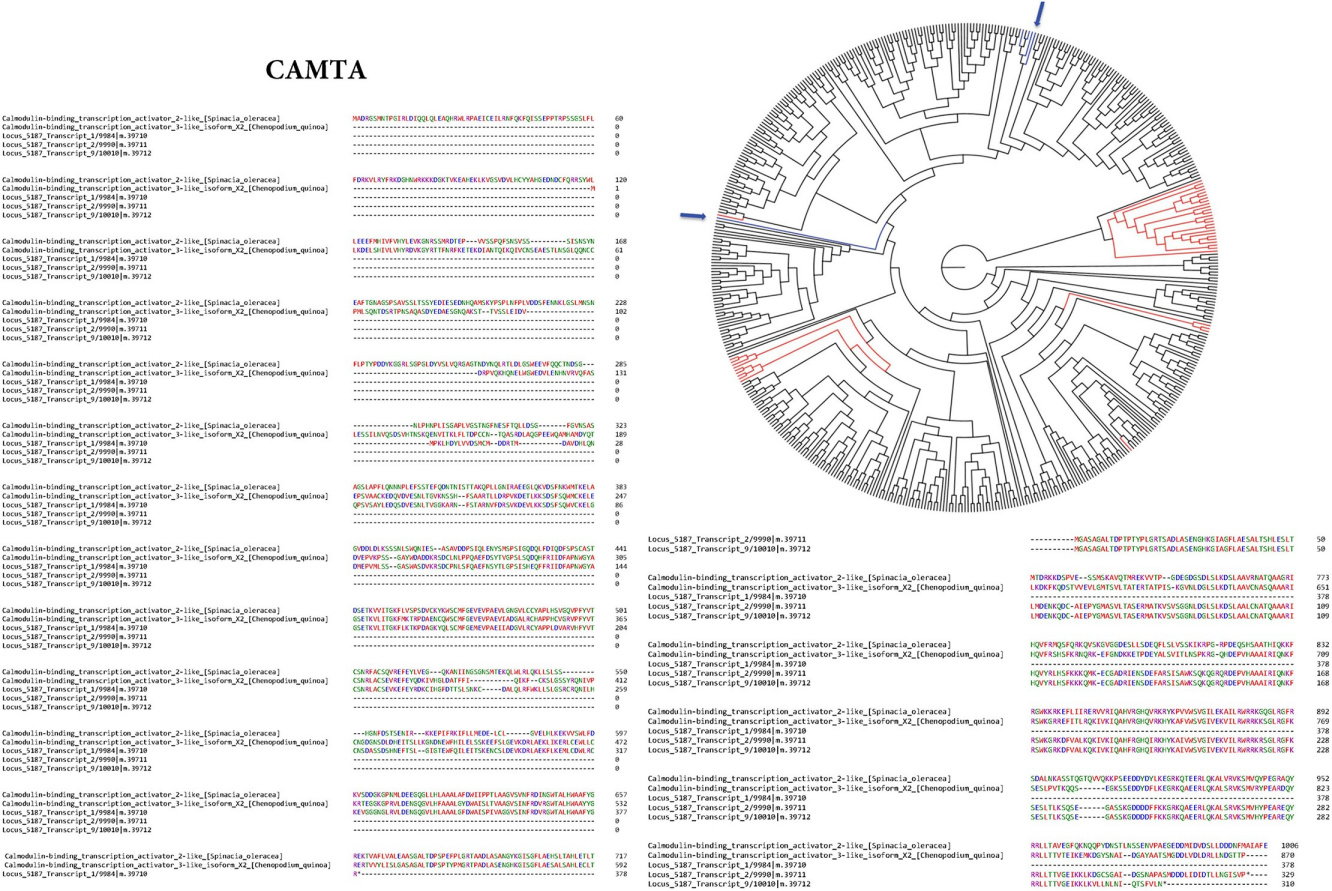
Multiple sequence alignment of CAMTA TFs. A BLAST search of CAMTA proteins from *S*. *fruticosa* was used to identify similar CAMTA proteins in *Spinacia oleracea* and *Chenopodium quinoa*. Amino acid sequences were aligned using the Clustal Omega server. Similarly classified residues are represented with the same color. Conserved residues are labeled with asterisks. A cladogram tree generated from the CAMTA TFs is also illustrated. The evolutionary tree includes CAMTA family TFs of green plants identified from PlantTFCBv.3.0, including *S*. *fruticosa* TFs of the family. Lines highlighted in red represent the total *S*. *fruticosa* TFs while blue lines represent the *S*. *fruticosa* TFs that are differentially expressed (locations marked by arrows).

We previously reviewed transcription factors identified in various halophytes that activate genes involved in cell maintenance, modifications and stress responses [8]. In this current work we performed protein sequence comparisons of *S*. *fruticosa* TF to known TF of other green plant species using NCBI BLAST (Table 1). Analysis of the amino acid sequence alignment of the *S*. *fruticosa* TFs against known *Spinacia oleracea* and *Chenopodium quinoa* TFs shows conserved sequences throughout the four families tested (S1, S2 and S3 Figs). We found that *S*. *fruticosa* CAMTA TF are related to Calmodulin-binding transcription activator 2-like protein in *Spinacia oleracea* and Calmodulin-binding transcription activator 3-like protein in *Chenopodium quinoa* (Fig 2). We found that *S*. *fruticosa* BZIP TF are related to BZIP TF 16-like isoform X2 in *S*. *oleracea and* BZIP TF 16-like protein in *C*. *quinoa* (S1 Fig). *S*. *fruticosa* MYB TFs are related to various MYB and LHY isoforms in both *S*. *oleracea* and *C*. *quinoa* (S2 Fig). *S*. *fruticosa* M-Type TF are related to MADS-box protein AGL24-like protein in both *S*. *oleracea* and *C*. *quinoa* (S3 Fig). These comparisons indicated that the TFs in *S*. *fruticosa* were correctly identified.

A total of 47,500 protein sequences from open reading frame (ORF) annotation of the *S*. *fruticosa* transcriptome were mapped against 57 TF families (MYB and MYB-related combined) from PlantTFDBv3.0 containing 129,288 TF from 83 species of green plants that have been comprehensively annotated with their functional domains, 3D structures, and gene ontology from various databases. Our analysis resulted in the identification of 3,110 TF across the different TF families. The TF assignments are summarized together with the percentage of TF family distribution (Table 2).

**Table 2:** Summary of transcription factor families.

TF family	Total	Percentage (%)	TF family	Total	Percentage (%)
FAR1	177	8.18	GRAS	25	1.16
bHLH	142	6.56	HSF	25	1.16
MYB	134	6.19	SBP	24	1.11
RAV	117	5.41	Dof	20	0.92
ARF	86	3.97	LBD	19	0.88
AP2	84	3.88	GRF	16	0.74
ERF	80	3.7	TCP	15	0.69
B3	79	3.65	NF-YB	14	0.65
HB-other	79	3.65	S1Fa-like	14	0.65
ARR-B	76	3.51	NF-YA	12	0.55
bZIP	71	3.28	CPP	11	0.51
NAC	70	3.23	WOX	10	0.46
MIKC	63	2.91	ZF-HD	9	0.42
C3H	57	2.63	NF-YC	8	0.37
M-type	57	2.63	SAP	8	0.37
WRKY	52	2.4	YABBY	8	0.37
C2H2	50	2.31	SRS	7	0.32
G2-like	50	2.31	NF-X1	5	0.23
CO-like	49	2.26	BBR-BPC	4	0.18
HD-ZIP	46	2.13	EIL	4	0.18
GATA	45	2.08	GeBP	4	0.18
CAMTA	44	2.03	LSD	4	0.18
HB-PHD	41	1.89	VOZ	4	0.18
Trihelix	31	1.43	E2F_DP	3	0.14
BES1	26	1.2	NZZ_SPL	3	0.14
Nin-like	26	1.2	STAT	2	0.09
TALE	26	1.2	Whirly	2	0.09
DBB	25	1.16	HRT-like	1	0.05
					
			Total	2164	100

The assignments of transcription factors to each family from PlantTFDBv.3.0 are summarized. This includes the percentage of distribution among the total TF families.

The results show that the most abundant TF family in *S*. *fruticosa* belongs to FAR1 with 177 identified TFs (8.18%). TF family bHLH is the next highest with 142 members (6.56%), followed by MYB with 134 TF (6.19%) and RAV as the fourth most abundant with 117 TF (5.41%). The smallest family belongs to HRT-like with only one hit. No TFs from the LFY gene family were found. These abundant TF are likely involved in other functional and structural mechanisms in the plant rather than salinity stress responses.

Although the FAR1 family has the highest number of identified TFs in *Suaeda*, a different pattern was observed when differentially expressed (DE) genes were quantified. No FAR1 TFs were differentially expressed between the tested salt treatments. This suggests that the FAR1 TF family might have a different function rather than long-term salinity stress regulation. The bHLH family is the second highest in abundance with two DE bHLH TF between long-term no salt and optimum salt treatment in *S*. *fruticosa*. The MYB TF family was the third highest in abundance in our analysis. RAV is the fourth most abundant TF family identified in this study with two DE genes.

### Evolutionary analysis of transcription factor encoding genes in *Suaeda fruticosa*

**Fig 3:**
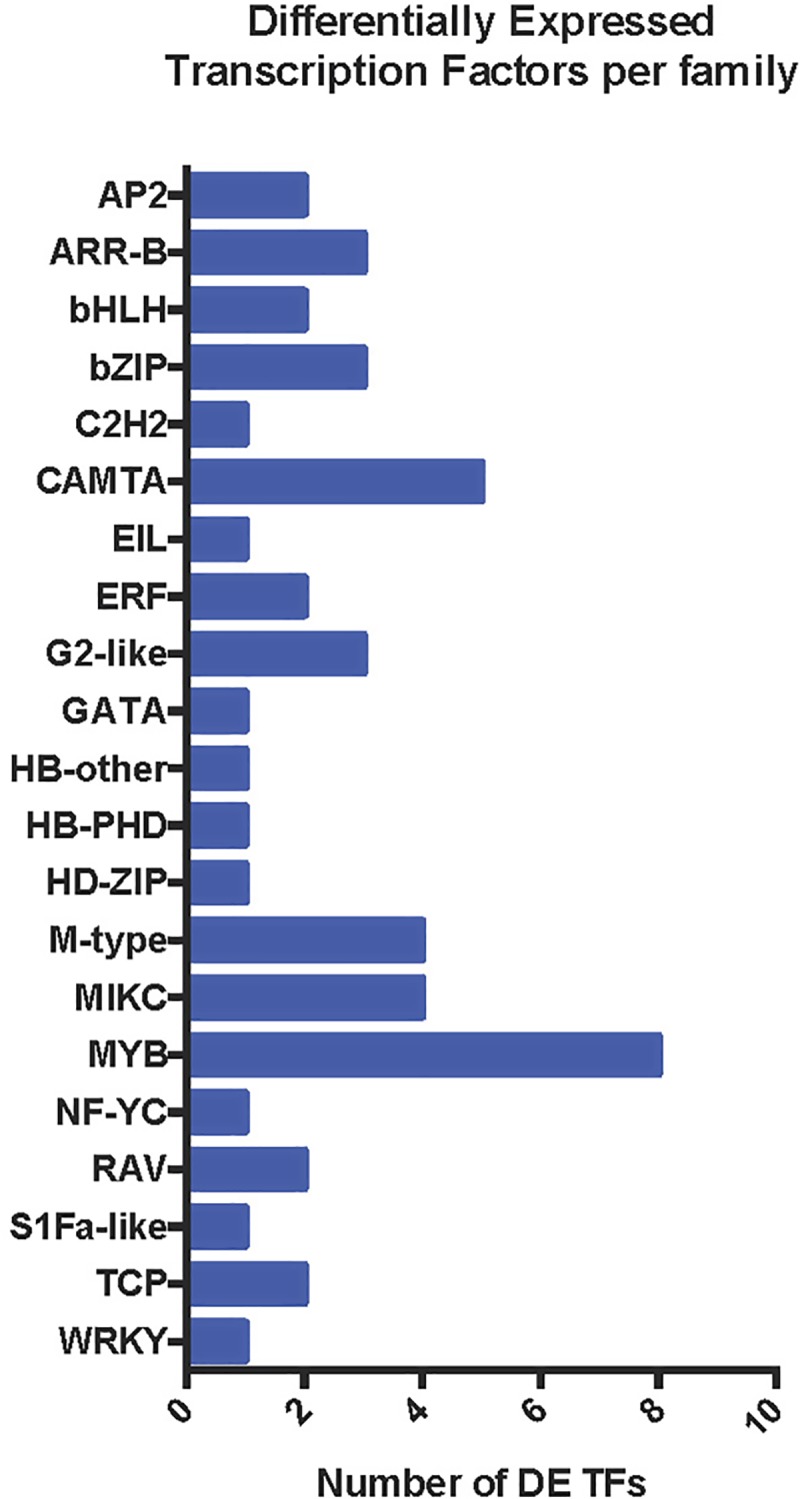
Summary of differentially expressed transcription factors in *S*. *fruticosa*. Numbers of differentially expressed transcription factor (DE TF) genes are shown.

We reconstructed 57 ML TF family trees using the iterative alignment-tree searching algorithm in PASTA (Fig 3). The CAMTA TF family tree shows that the majority of DE and non-DE TF genes formed single monophyletic clades (Fig 2) [19, 20]. In the bZIP TF family, most of the DE and non-DE TF genes were scattered uniformly across the tree; however, all four DE genes formed a single monophyletic cluster (Fig 2 and S4 Fig). Such distribution of bZIP genes suggests that gene duplications happened before speciation of *S*. *fruticosa*.

The M-type tree (a subset of MADS-box) also exhibits similar relationships between DE and non-DE TF genes (S4 Fig). Nevertheless, all four M-type DE genes cluster with non-DE genes, suggesting their recent adaptive radiation as a response to salt. For the MYB TF family we observed similar patterns where four DE genes formed a monophyletic group whereas non-DE genes were uniformly distributed across the tree (S4 Fig).

### Identification and annotation of differentially expressed transcription factor genes

We have focused on salt-responsive transcription factors that are differentially expressed (DE) between long-term contrasting conditions (no salt 0 mM NaCl versus optimum salt 300 mM NaCl concentration) with plants grown in a growth chamber. We performed differential expression analysis of the *S*. *fruticosa* transcriptome using EdgeR. The method compares significant transcript expression levels between specific treatments following a negative binomial model using the Benjamini-Hochberg method for multiple testing correction at a false discovery rate cutoff of 0.05 [21]. We identified 49 DE TF using a pHMM search against TF family databases from PlantTFDBv.3.0. The summary of DE TF identified that the greatest number belong to the MYB superfamily (MYB and MYB-related) with 8 TF members, CAMTA with 5, and MIKC and M-type (both MADS box family) with 4 TF. bZIP, ARR-B and G2-like families all have 3 TF members in this analysis (Fig 3).

**Fig 4:**
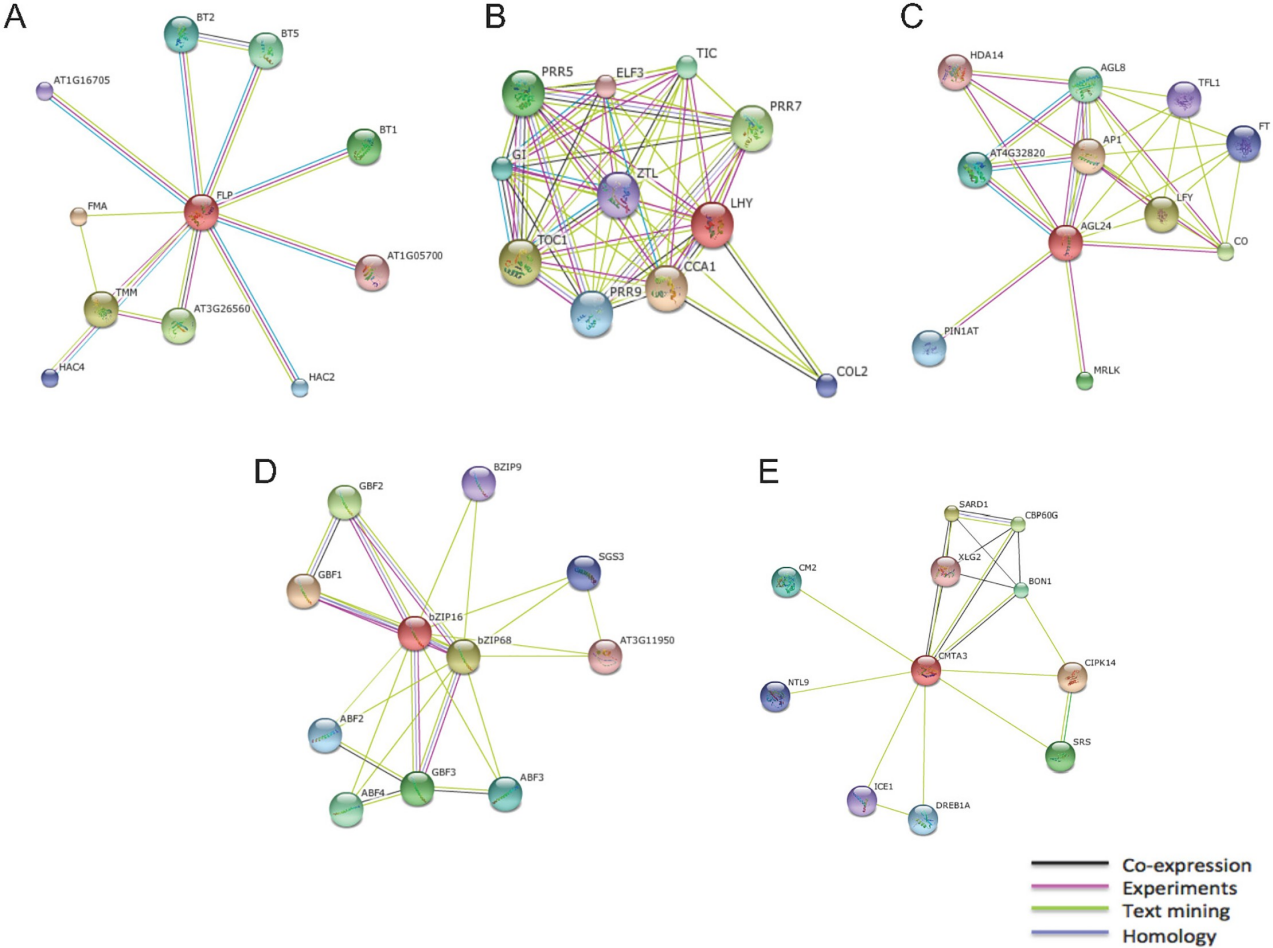
Protein-protein interaction networks of *S*. *fruticosa* transcription factors predicted by STRING. Interactions of selected DE TFs from the top DE families are illustrated: MYB TF FLP (A), MYB TF LHY and CCA1 (B), MADS-box AGL24 and LFY (C), bZIP family bZIP16 and bZIP 68 (D), CAMTA family CMTA3 (E). Colored lines represent different interactions: black (co-expression), pink (experimental-based on analysis of available database information), green (text mining), and blue (homology).

We chose the top 4 DE TF families (MYB, CAMTA, MADS-box and bZIP) for expression profiling, phylogenetic tree construction and gene ontology annotation by analysis of available information from the databases (Fig 4). The MYB superfamily contains the highest number of DE TF between treatments and is the third most abundant TF family (Table 2 and Fig 3) found in *S*. *fruticosa*.

### Protein interaction network of differentially expressed transcription factors

The summary network of predicted physical and functional interactions among the identified DE TFs suggests involvement in flowering, stomatal development and stress regulation (S5 Fig). Importantly, the protein relationships predicted in *S*. *fruticosa* using Arabidopsis homologs MYB (FLP1, MYB13, LHY), ARR-B PCL1, and MADS-box AGL24 are involved in the same interaction network.

Genes that belong to the top DE TF families were also examined for their predicted interactions and potential functions with other genes (Fig 4). From the identified interactions between DE TFs, two *S*. *fruticosa* genes (Locus_17372_Transcripts_9,12), encoding similar identity with FLP (88% identity) and MYB88 (79% identity), contain a putative MYB transcription factor involved in stomata development (Fig 4A). The loss of FLP activity results in failure of guard mother cells to adopt the guard cell fate [22]. FLP and MYB88 negatively control the expression of genes associated with stomatal development but positively regulate gene expression related to stress conditions. Double mutants of FLP and MYB88 are more susceptible to drought and salt stress and lose water significantly faster than wild-type [23]. This suggests that these individual TFs may play important roles in salt regulation.

Four DE genes (Locus_36812_Transcript_1,2,5,6) related to LHY or CCA1 are predicted to interact with other MYB TFs (Fig 4B). CCA1 regulates ELF4 and ELF3 that are involved in circadian control and phytochrome regulation in C3 and CAM leaves [24]. The DE MADS-box AGL24 (Locus_82944_Transcripts_1,3,4,6) homologue is also predicted to interact with these MYB homologs (Fig 4C). These clock-associated genes in *Mesembryanthemum* are unaffected by salt stress, suggesting compensation of the central circadian clock against development and abiotic stress in specialized plants [25].

Other families, including three *S*. *fruticosa* bZIP16 homologs (Locus_50829_Transcript_4,7,8), interact with ABF genes and other bZIP genes (Fig 4D). Arabidopsis bZIP16 promotes seed germination and hypocotyl elongation during early stages of seedling development. CAMTA3 homologues (Locus_5187_Transcript_1/9984, 1/9985, 1/9988, 2,9) show interactions with DREB dehydration response elements, regulators of cell death and defense, and other genes important to regulation of plant immunity (Fig 4E).

Sequences identified from the BLAST and SwissProt databases were mapped with GO terms and assigned functional terms based on the gene ontology vocabulary (S6 Fig). The TF are assigned into three main categories: Biological process refers to the biological objective of the genes or gene products, molecular function as the biochemical activity of the genes, and cellular components as the place where the interaction of the gene product actively functions. Dominant categories include metabolic, developmental and single organism process and stimulus response (each comprising 9%) for biological processes (S6A Fig). There are 59 hits (31%) for general binding for the molecular function category (S6B Fig), and cellular component category shows 28% of hits for cell part and organelle where the interaction of the genes is happening (S6C Fig). This annotation of *S*. *fruticosa* DE TFs suggests that they are involved in salt regulation but may likely also perform diverse functions in other regulatory, metabolic and stress response mechanisms.

### Validation of DE by quantitative reverse-transcriptase PCR (qRT-PCR) analysis

**Fig 5:**
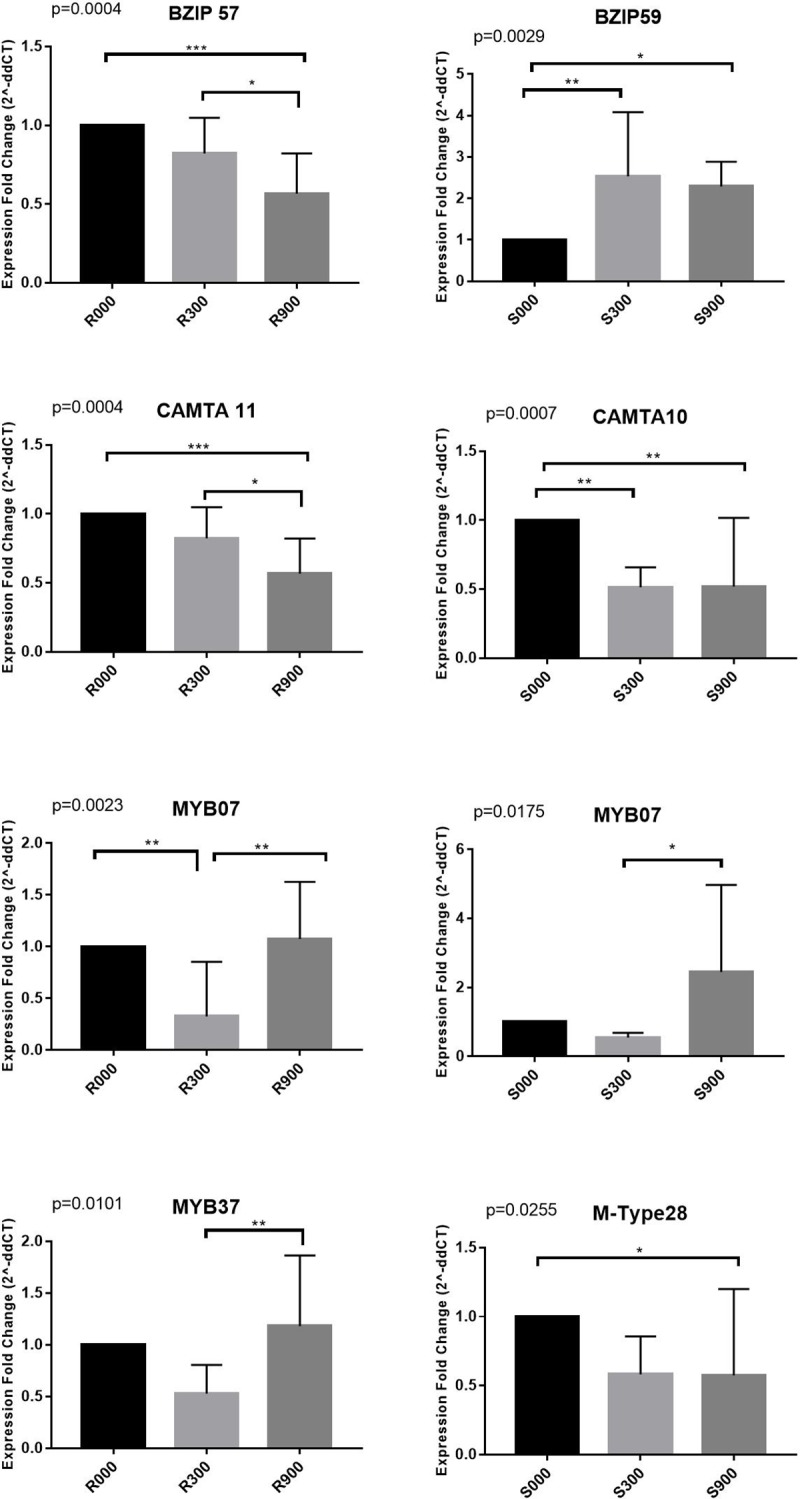
qRT-PCR validation of differentially expressed transcription factor genes in *S*. *fruticosa*. Fold changes in expression were calculated using alpha tubulin as the endogenous control. Standard error of the mean was calculated using the Prism Graph Pad data analysis package. R000 (roots at 0 mM NaCl), R300 (roots at 300 mM NaCl), R900 (roots at 900 mM NaCl), S000 (shoots at 0 mM NaCl), S300 (shoots at 300 mM NaCl), S900 (shoots at 900 mM NaCl).

To validate the results from the transcriptome analysis, we selected DE genes in each of the top four families (bZIP, CAMTA, MYB and M-Type) for quantitative reverse transcriptase PCR (qRT-PCR) analysis to measure gene expression among different treatments and tissue types (roots and shoots). Specific primers were optimized for the selected TF genes using alpha tubulin as an endogenous control (S1 File). We amplified cDNA libraries from three biological replicates of roots and shoots for 0 mM and 300 mM treated plants. Several of the tested gene targets showed similar changes in gene expression (Fig 5) to those observed in the transcriptome analysis [11]; e.g. M-Type 26 and 28 (MADSbox AGL24) genes, which are downregulated at 300mM NaCl concentration. In addition, in this current analysis we included analysis of differential gene expression in plants grown in 900 mM NaCl, which significantly inhibits growth of *S*. *fruticosa*. Statistically significant decreases in expression of bZIP57 are observed in the 300 mM treated shoots, which correspond closely with the RNA-sequencing results. Similarly, there is a decrease of CAMTA12 expression in the 300 mM shoots. MADSbox29 shows a significant decrease of expression in shoots in optimal growth conditions of 300 mM NaCl, while MYB72 shows upregulation on the same tissue type and treatment.

## Discussion

To identify and characterize putative transcription factors (TFs) in an obligate halophyte, *Suaeda fruticosa*, we utilized the RNA-seq data published earlier [11] to identify and characterize putative TFs that are potentially involved in salt tolerance. We analyzed the expression patterns of specific TF families, protein-protein interactions and evolutionary trajectories to predict the roles of differentially expressed TFs in salt tolerance. TF families with the most differentially expressed (DE) genes in response to salinity were identified as members of the MYB, CAMTA, MADS-box and bZIP families.

The FAR1 family has the highest number of identified TFs in *S*. *fruiticosa*, but none were observed to be differentially expressed between the tested salt treatments. This suggests that the FAR1 TF family is likely not involved in long-term salinity stress regulation but rather has other functions in the plant. As one possible example, Arabidopsis FAR1 TFs have been reported to bind to promoters of abscisic acid (ABA) genes to activate expression. In particular, under salt and osmotic stress, FAR1 has been shown to trigger the accumulation of ABA [26]. When FAR1 genes lose their functionality (e.g. by deletion), sensitivity to ABA-mediated inhibition of seed germination is reduced. Also, FAR1 member fhy3 and far1 mutants exhibit wider stomata, lose water faster, and are more sensitive to drought [27].

The second highest number of TFs identified were of the bHLH family. BHLH TFs are involved in salt stress tolerance and developmental processes in tobacco [28] and rice [29, 30]. However, there are limited halophyte studies focusing on the involvement of bHLH TFs in drought and salinity stress in halophytes [31, 32]. Overexpression of some bHLH genes were found to confer increased tolerance to salt and osmotic stress in Arabidopsis. This TF family has been observed to positively regulate salt-stress signals independent of ABA, and have been targets to improve salt tolerance in crops [33].

MYB TFs were the third highest in abundance, and are known to operate through ABA-dependent or independent pathways. Among genome-wide identification and expression analyses related to plant abiotic stress, MYB is one of the most studied TF families in halophytes [31, 34]. It has been suggested that following duplication events, MYB TFs often undergo sub-functionalization [35]. The MYB TF family is involved in controlling various cellular processes, including several abiotic and epigenetic control of stress responses [36]. This is consistent with our findings that *S*. *fruticosa* roots at 300 mM salt conditions show downregulated expression of MYB 07 and MYB 37, while expression is upregulated under stress conditions (no salt 000 mM and 900 mM NaCl treatments.)

MYB plays diverse physiological and developmental roles that are either induced or repressed under different stress conditions [6]. In Arabidopsis, MYB2 is induced by salt and drought stress. Rice OsMYB2 encodes a stress-responsive MYB that plays a regulatory role in salt, cold and dehydration [37]. In the halophyte *Avicennia marina*, the AmMYB1 gene confers increased salt tolerance with reduced chlorosis and other salt stress symptoms when introduced to tobacco plants [38]. The DE FLP and MYB88 putative MYB transcription factors may be involved in stomata development. The loss of FLP activity results in failure of guard mother cells to adopt the guard cell fate [22]. FLP and MYB88 negatively control the expression of genes associated with stomatal development but positively regulate gene expression related to stress conditions. Double mutants of FLP and MYB88 are more susceptible to drought and salt stress and lose water significantly faster than wild-type [23]. This suggests that these individual TFs may play important roles in salt regulation. These findings suggest that the MYB TF family in *S*. *fruticosa* is the most likely key transcription regulator for salt tolerance regulation.

Several differentially expressed M-type MADS-box genes, the third most abundant group, were identified in our study. The ancestral functions of MADS-box genes are currently unknown. Comparison of some MADS-box genes in Arabidopsis showed that they are polyphyletic with significantly longer branch lengths than for other genes, suggesting that they could be pseudogenized as a result of neutral evolution [39]. Most likely these copies appeared via whole genome duplications and intraspecific gene duplications [39, 40].

The RAV TF family was found to have two DE genes in *S*. *fruticosa*. Some members of the RAV family have been found to modulate drought and salt-stress responses in Arabidopsis and are involved in ethylene and brassinosteroid responses [41].

Some genes that were selected from each TF family for qRT-PCR analysis showed statistically significant differential gene expression (Fig 5), while others did not (S7 Fig). This suggests that not all of the TFs tested are strongly linked to salt stress, and some may be involved in other pathways such as maintenance of the homeostatic balance in the plant. Four MADS-box DE genes were identified in *Suaeda* upon salt treatment. MIKC and M-type TFs show similar gene hits since both belong to the same MADS-box TF family. MIKC type TFs contain a keratin-like coiled-coil (K) domain while M-type lacks this domain. MADS-box family TF genes are involved in fruit development, seed pigmentation, floral organ identity determination, and stress response in several species [42]. MADS-box family TFs are potential candidates for salt regulation in *S*. *fruticosa*. In *Brassica rapa*, several MADS-box family TFs were shown to be induced by cold, drought and salt stresses [43]. In rice, three genes (OsMADS2, 30 and 55) showed more than 2-fold downregulation in response to dehydration and salt stress [44]. We also investigated another part of the MADS-box family, the M-type TFs, involved in flowering and reproduction organ development. This likely explains that we cannot adequately compare expression levels in the root tissue because these TFs are most likely not expressed in the roots [45].

Whole genome/large-scale chromosomal duplications play a crucial role in increasing copy number of CAMTA TF genes [20]. The close-relatedness of DE TF paralogs found most likely indicates that these genes were duplicated separately from other non-DE TFs, and subsequently their expression patterns and regulatory mutations were preserved by species-specific environmental constraints related to increased salt concentration [19]. Based on observed patterns, we hypothesized that such large CAMTA family expansions can be explained by small-scale gene duplication events (e.g. via unequal crossing over).

There were three differentially expressed bZIP TFs identified upon salt treatment in *S*. *fruticosa*. The BZIP TF family regulates light responsive genes and abscisic acid (ABA) mediated abiotic stress signaling pathways [46], and involves binding to G-box motifs [47]. The group F bZIP family from Arabidopsis and its related halophyte species was identified to be a key regulator of salt stress adaptation [48]. Arabidopsis AREB1, AREB2 and ABF3 are also important genes for signaling under drought stress. Group A bZIP in rice and tomato confers increased tolerance to water deficit and salt stress [49]. The *S*. *fruticosa* bZIP family is related to bZIP16, which acts as a repressor of LHCB2.4 [50]. In the shoots of *S*. *fruticosa*, we found that BZIP 59 is downregulated in 0 mM salt conditions compared to 300 mM. Similarly, 900 mM salt treated roots also have downregulated BZIP59 compared to 300 mM salt condition. The results with BZIP59 suggest that this TF may play a role in the optimal growth of *S*. *fruticosa* at 300 mM NaCl, as it is downregulated in both the no salt and elevated salt plants.

One of the major bZIP family expansions was observed on the branch that leads to seed plants[51]. Moreover, its evolution-by-gene duplication patterns fit to a random birth-death-model, suggesting that new gene copies occurred as a result of small-scale duplication events rather than whole genome/chromosome duplications [51].

Protein interaction predictions identified a number of potential important interactions involved in salinity tolerance. The AGL24 transcriptional activator mediates effects of gibberellins on flowering and regulates the expression of LFY genes for floral induction and development. A homologue of MYB13 (Locus_37251_Transcript_2) is involved in response to salt stress, jasmonic acid and gibberellin [25] and interacts with homologue PCL1 (Locus_119717_Transcript_1,2). PCL1 works as a transcriptional activator involved in circadian rhythm and regulation of flower development in Arabidopsis [52]. CAMTA3 was predicted to interact with DREB dehydration response elements. Studies of CAMTA3 in other plants reveal that it negatively regulates plant defense and suppresses salicylic acid accumulation and disease resistance. Calcium ion/calmodulin binding through CAMTA3 is critical for wound response. Overexpression of AtSR1/CAMTA3 effectively confers plant resistance to herbivore attack through salicylic acid/jasmonic acid crosstalk regulation [53, 54].

## Conclusions

In conclusion, we have identified several differentially expressed transcription factor genes in *S*. *fruticosa*, conducted phylogenetic analysis for top DE TFs, performed expression pattern analysis, and annotated predicted individual TFs involved in interaction networks. Phylogenetic analysis showed that the observed DE TFs are strongly conserved across plant species. This builds upon the very limited available information on TFs in succulent halophytes. Only minimal information on succulent halophytes is available [55], although considerable work has been done to identify transcription factors in non-succulent halophytes [56]. The results presented here provide basic information on key regulator TFs of *S*. *fruticosa* and contribute to an increased understanding of salt tolerance mechanisms of a succulent halophyte that may be utilized for the improvement of halophytes as non-conventional crops. Future analyses should include individual examination of the transcription factors identified in relation to salt tolerance between halophytes and salt-sensitive glycophytes.

## Supporting information

S1 File Primers designed for qRT-PCR. (DOCX)

S1 Fig Multiple sequence alignment of BZIP. A BLAST search of BZIP proteins from *S*. *fruticosa* were used to determine similar BZIP proteins in *Spinacia oleracea* and *Chenopodium quinoa*. Amino acid sequences were aligned by the Clustal Omega server. Similarly classified residues are represented with the same color. Conserved residues are labeled with asterisks.
(TIF)

S2 Fig Multiple sequence alignment of MYB. A BLAST search of MYB proteins from *S*. *fruticosa* were used to determine similar MYB proteins in *Spinacia oleracea* and *Chenopodium quinoa*. Amino acid sequences were aligned by the Clustal Omega server. Similarly classified residues are represented with the same color. Conserved residues are labeled with asterisks. A separate alignment was performed for each (a) MYB07, (b) MYB37, and (c) MYB72 because these three proteins show high variation.
(TIF)

S3 Fig Multiple sequence alignment of M-Type. A BLAST search of M-Type proteins from *S*. *fruticosa* were used to determine similar M-Type proteins in *Spinacia oleracea* and *Chenopodium quinoa*. Amino acid sequences were aligned by the Clustal Omega server. Similarly classified residues are represented with the same color. Conserved residues are labeled with asterisks.
(TIF)

S4 Fig Cladogram trees from BZIP, MADS-box, and MYB TFs. Evolutionary trees include TFs of green plants identified from PlantTFDBv.3.0 belonging to the respective TF family and identified *S*.*fruticosa* TFs of that family. Red highlighted lines represent the total *S*. *fruticosa* TFs while blue lines represent those *S*. *fruticosa* TFs that are differentially expressed. Arrow indicates the DE TFs locations.
(TIF)

S5 Fig Protein interaction network of differentially expressed transcription factors in *S*. *fruticosa*. Each node represents a protein and each edge represents interaction, colored by evidence type. Input includes homologous sequence from Arabidopsis: LHY, MYB13, FLP, WAKL2, AT1G16260, RAP2.12, IDD7, AT1G68920, WRKY57, EIL3, CMTA3, HB6, RR12, AT2G26730, bZIP16, ACR6, NF-YC11, RAP2.2, PERK1, PCL1, AT3G57750, GATA26, AGL24, BSK1, AT5G21090, AT5G23280. MOL1, AT5G64220.
(JPG)

S6 Fig Gene Ontology Summary of total assembled ESTs using BLAST2GO. Distribution of Gene Ontology Annotation of the *Suaeda fruticos*a transcriptome. The results are summarized as follows: (A) Biological Process, (B). Cellular component (C) Molecular Function.
(TIF)

S7 Fig qRTPCR validation of the transcriptome data. Each graph shows the qRTPCR results for test genes. The annotated putative genes are titled and the mean fold change represented by the 2-ΔΔCT method relative to 0 mM treated samples are shown on the y axis. Error bars depict the standard error of the mean for 3 biological replicates. R000 (roots at 0 mM NaCl), R300 (roots at 300 mM NaCl), R900 (roots at 900 mM NaCl), S000 (shoots at 0 mM NaCl), S300 (shoots at 300 mM NaCl), S900 (shoots at 900 mM NaCl).
(TIF)

## Abbreviations

TF: transcription factor
DE: differential expression/differentially expressed
GO: gene ontology
ML: maximum likelihood
