# Supplementary material for: Identification and evolutionary characterization of salt-responsive transcription factors in the succulent halophyte Suaeda fruticosa
Source: PLoS One. 2019 Sep 23;14(9):e0222940. doi: 10.1371/journal.pone.0222940 (PMC6756544; doi:10.1371/journal.pone.0222940)
Supplement: S1 File — (DOCX) [file pone.0222940.s001.docx]

**Primers designed for qRTPCR**

MYB37 FWD

CAT GAG GAT GTC GGA GCA TTA T

MYB37 REV

GTT GCA CAG GAC AGG AAT TTG

MYB72 FWD

AGG AAC CTG ATG CTG ATG ATG

MYB72 REV

CAG TGG AGG ATG GTG TTT CTT

MYB07 FWD

GAG GTG TTG TCC GTT GAA GA

MYB07 REV

GAA CGT CGT CCG ACA TAT ACA C

CAMTA10 FWD

GAA AGG CCA GGA ACT TCT CTA C

CAMTA10 REV

TGG CTC CAT GTC TCC TAA CT

CAMTA11 FWD

CCA TTA TCC AGA AGC GAG AGA G

CAMTA11 REV

CAT CAA TTG CGC CAC TAC AC

CAMTA12 FWD

CAA TCT GAG GGC GCT TCT T

CAMTA12 REV

GCT CTC TCG CTT CTG GAT AAT G

MADSbox26 FWD

CTT CTG GCA AAC TCC ATG ATT TC

MADSbox26 REV

GGA TCA AGC TGT TGA GGA AGA

MADSbox28 FWD

TTA AGC CGA ATG CTA GGA GAA G

MADSbox28 REV

GCT TGA GGT CTA CGA TCA CTT T

MADSbox29 FWD

CTT CTG GCA AAC TCC ATG ATT TC

MADSbox29 REV

GGA TCA AGC TGT TGA GGA AGA

bZIP57 FWD

GGA TGA CTA TGG TGC CAA TGA

bZIP57 REV

CGT ATA GCC TGG ATT GGA GAT G

bZIP59 FWD

CGT AGA TCC AGA CTG CGT AAA C

bZIP59 REV

GCC CTA AGC TGC TCG TAA TC

bZIP60 FWD

GGA TGA CTA TGG TGC CAA TGA

bZIP60 REV

CGT ATA GCC TGG ATT GGA GAT G

A tubulin FWD

CAC GCG CTG TAT TCG TAG AT

A tubulin REV

TGA CCA CGA GCG AAG TTA TTA G
