# Supplementary figures and images for: Identification and evolutionary characterization of salt-responsive transcription factors in the succulent halophyte Suaeda fruticosa
Source: PLoS One. 2019 Sep 23;14(9):e0222940. doi: 10.1371/journal.pone.0222940 (PMC6756544; doi:10.1371/journal.pone.0222940)

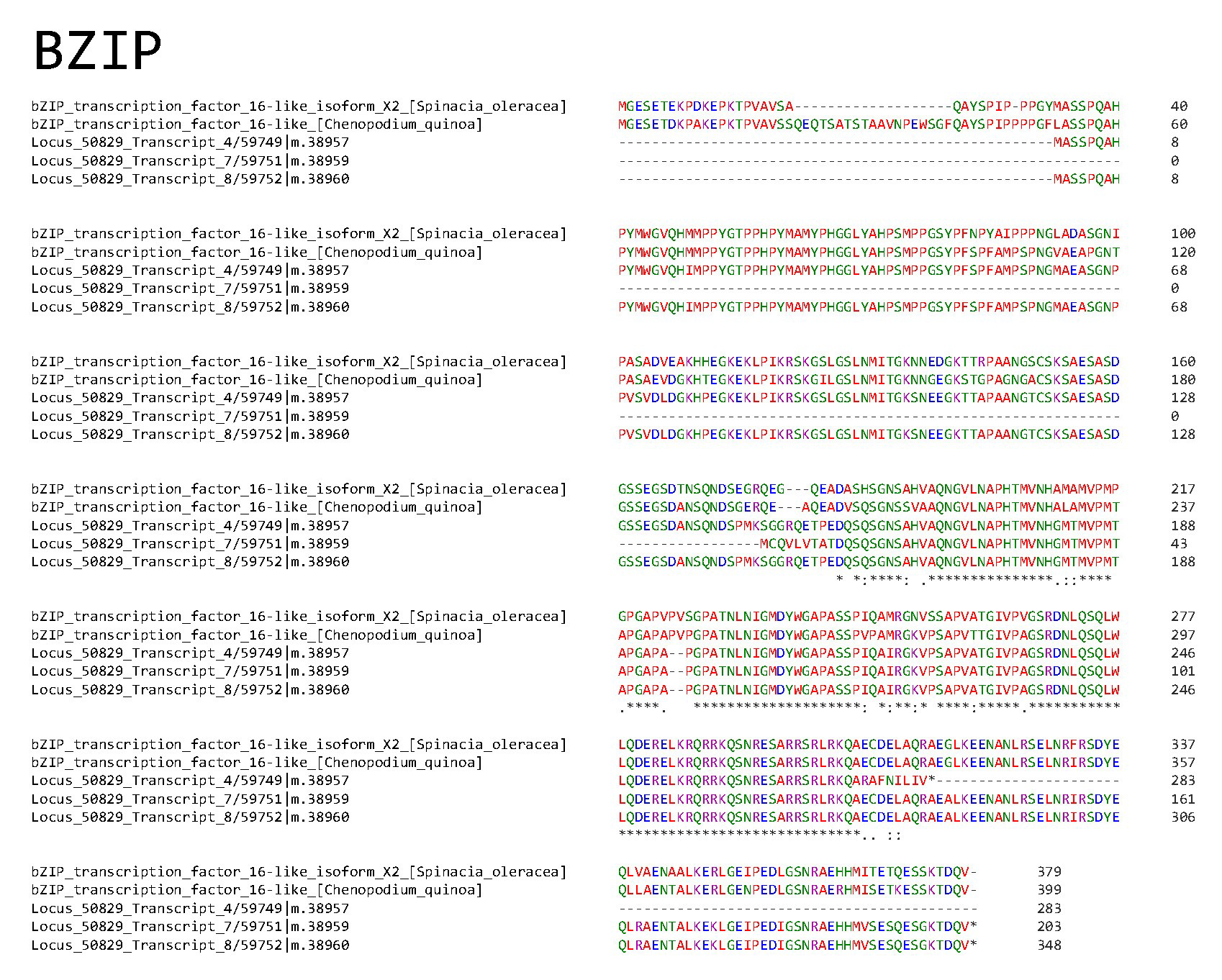

Supplement: S1 Fig — A BLAST search of BZIP proteins from S. fruticosa were used to determine similar BZIP proteins in Spinacia oleracea and Chenopodium quinoa. Amino acid sequences were aligned by the Clustal Omega server. Similarly classified residues are represented with the same color. Conserved residues are labeled with asterisks. (TIF) [file pone.0222940.s002.tif]

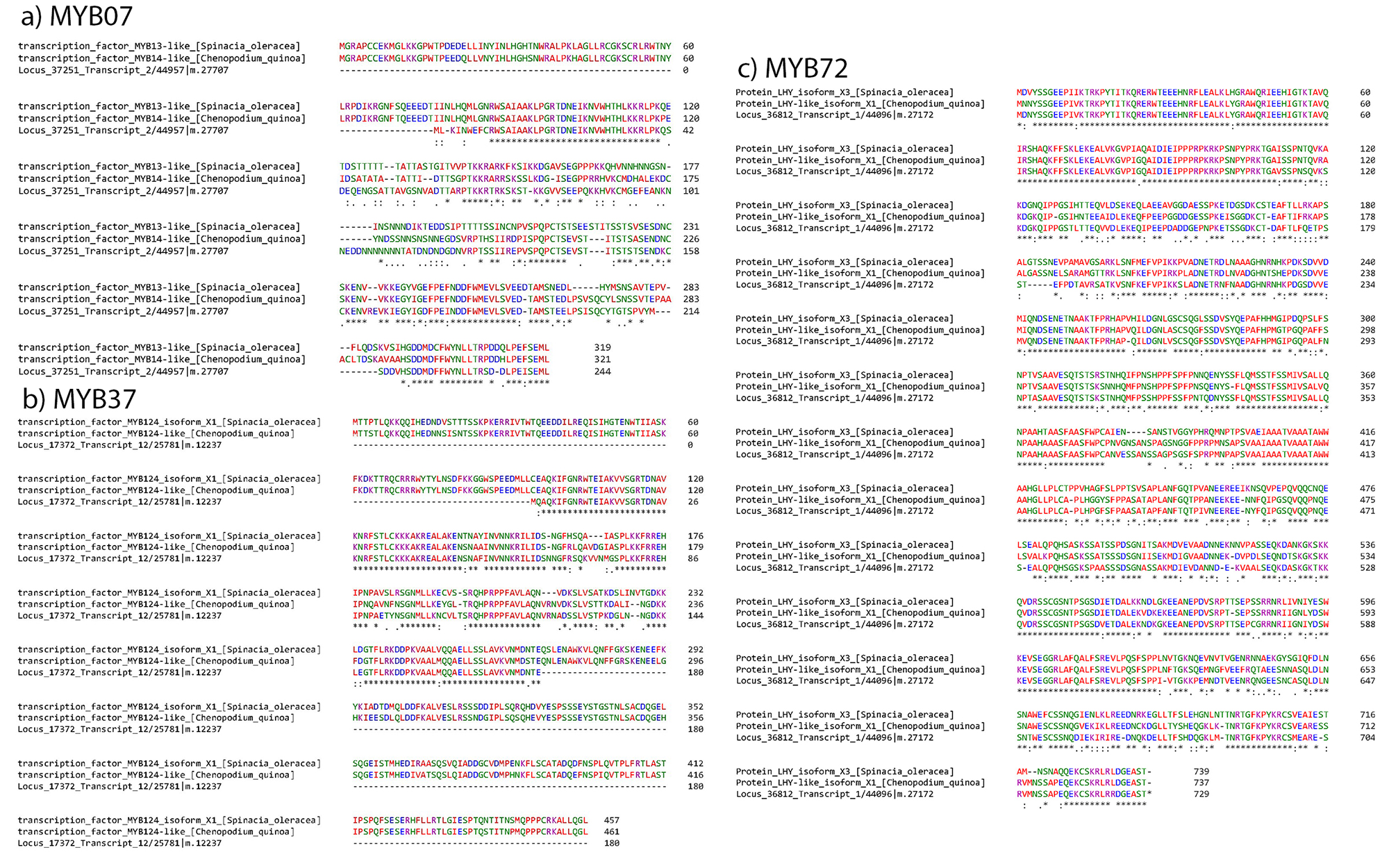

Supplement: S2 Fig — A BLAST search of MYB proteins from S. fruticosa were used to determine similar MYB proteins in Spinacia oleracea and Chenopodium quinoa. Amino acid sequences were aligned by the Clustal Omega server. Similarly classified residues are represented with the same color. Conserved residues are labeled with asterisks. A separate alignment was performed for each (a) MYB07, (b) MYB37, and (c) MYB72 because these three proteins show high variation. (TIF) [file pone.0222940.s003.tif]

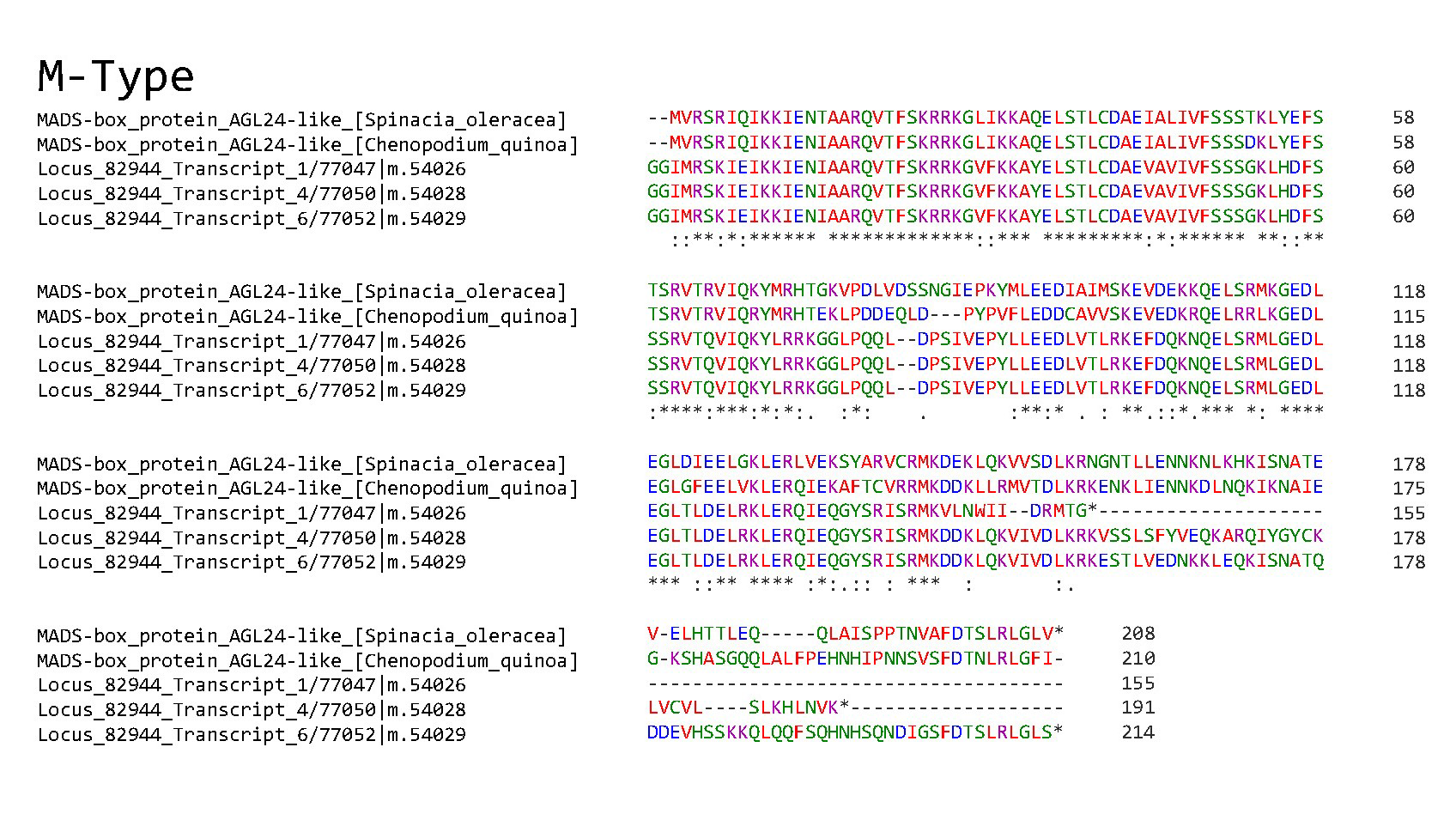

Supplement: S3 Fig — A BLAST search of M-Type proteins from S. fruticosa were used to determine similar M-Type proteins in Spinacia oleracea and Chenopodium quinoa. Amino acid sequences were aligned by the Clustal Omega server. Similarly classified residues are represented with the same color. Conserved residues are labeled with asterisks. (TIF) [file pone.0222940.s004.tif]

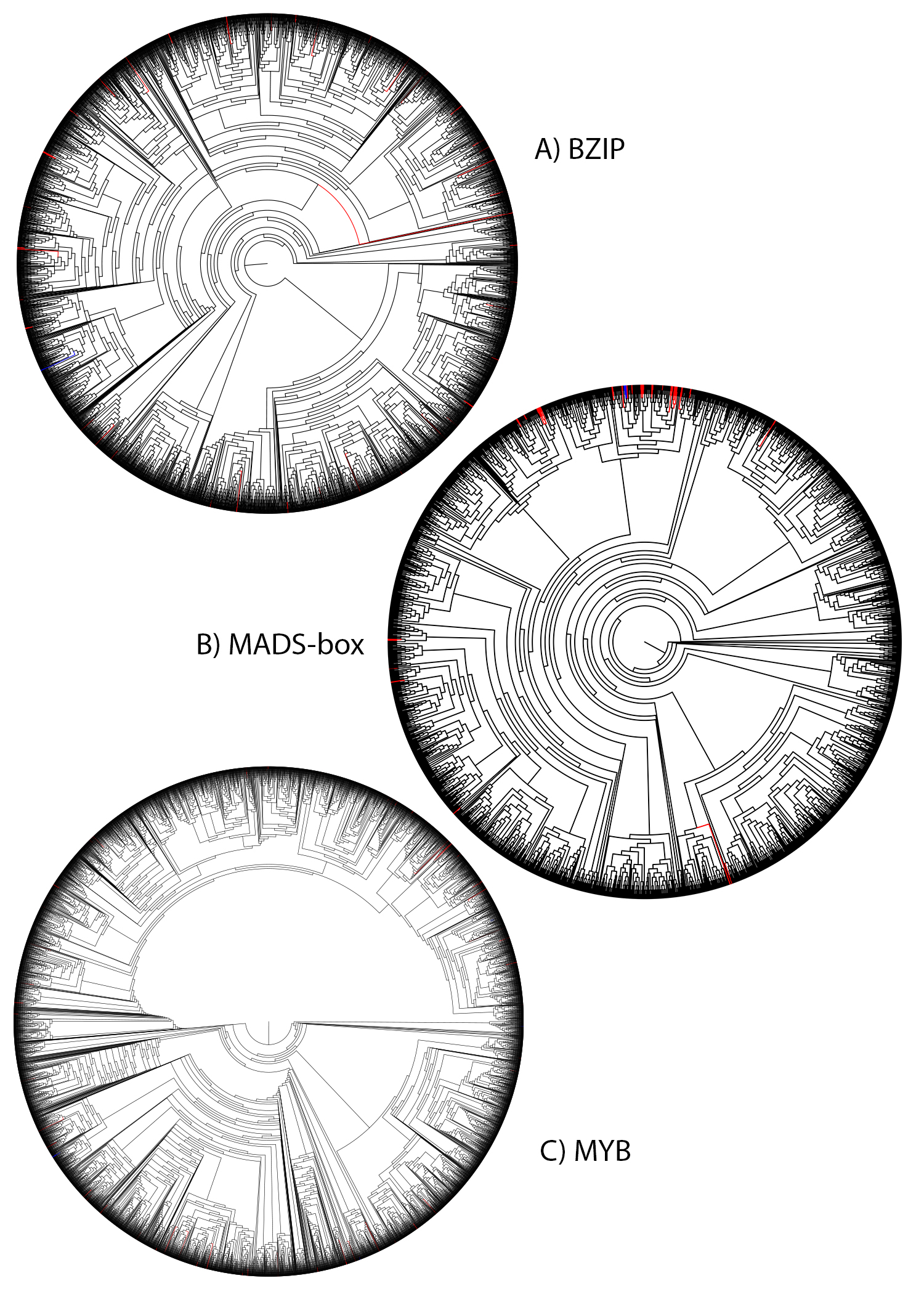

Supplement: S4 Fig — Evolutionary trees include TFs of green plants identified from PlantTFDBv.3.0 belonging to the respective TF family and identified S.fruticosa TFs of that family. Red highlighted lines represent the total S. fruticosa TFs while blue lines represent those S. fruticosa TFs that are differentially expressed. Arrow indicates the DE TFs locations. (TIF) [file pone.0222940.s005.tif]

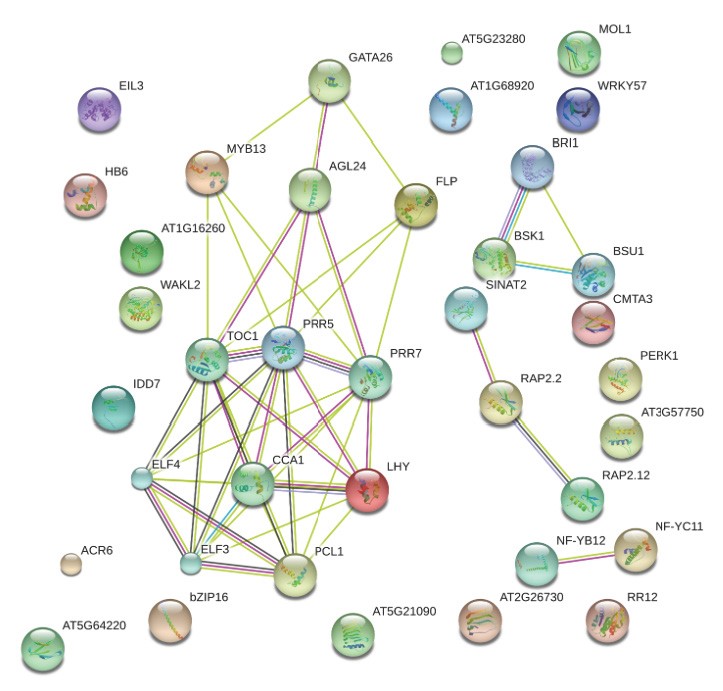

Supplement: S5 Fig — Each node represents a protein and each edge represents interaction, colored by evidence type. Input includes homologous sequence from Arabidopsis: LHY, MYB13, FLP, WAKL2, AT1G16260, RAP2.12, IDD7, AT1G68920, WRKY57, EIL3, CMTA3, HB6, RR12, AT2G26730, bZIP16, ACR6, NF-YC11, RAP2.2, PERK1, PCL1, AT3G57750, GATA26, AGL24, BSK1, AT5G21090, AT5G23280. MOL1, AT5G64220. (JPG) [file pone.0222940.s006.jpg]

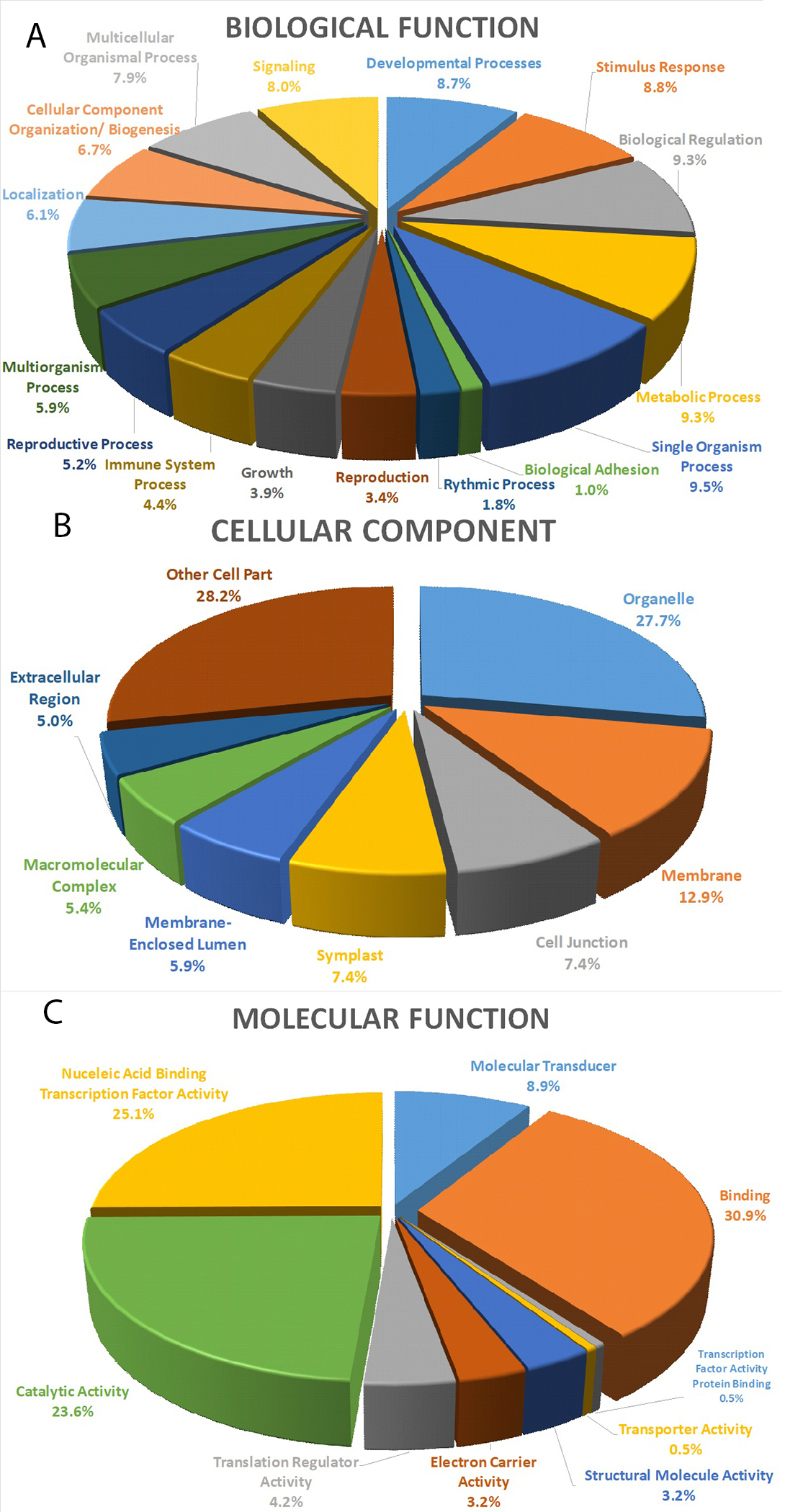

Supplement: S6 Fig — Distribution of Gene Ontology Annotation of the Suaeda fruticosa transcriptome. The results are summarized as follows: (A) Biological Process, (B). Cellular component (C) Molecular Function. (TIF) [file pone.0222940.s007.tif]

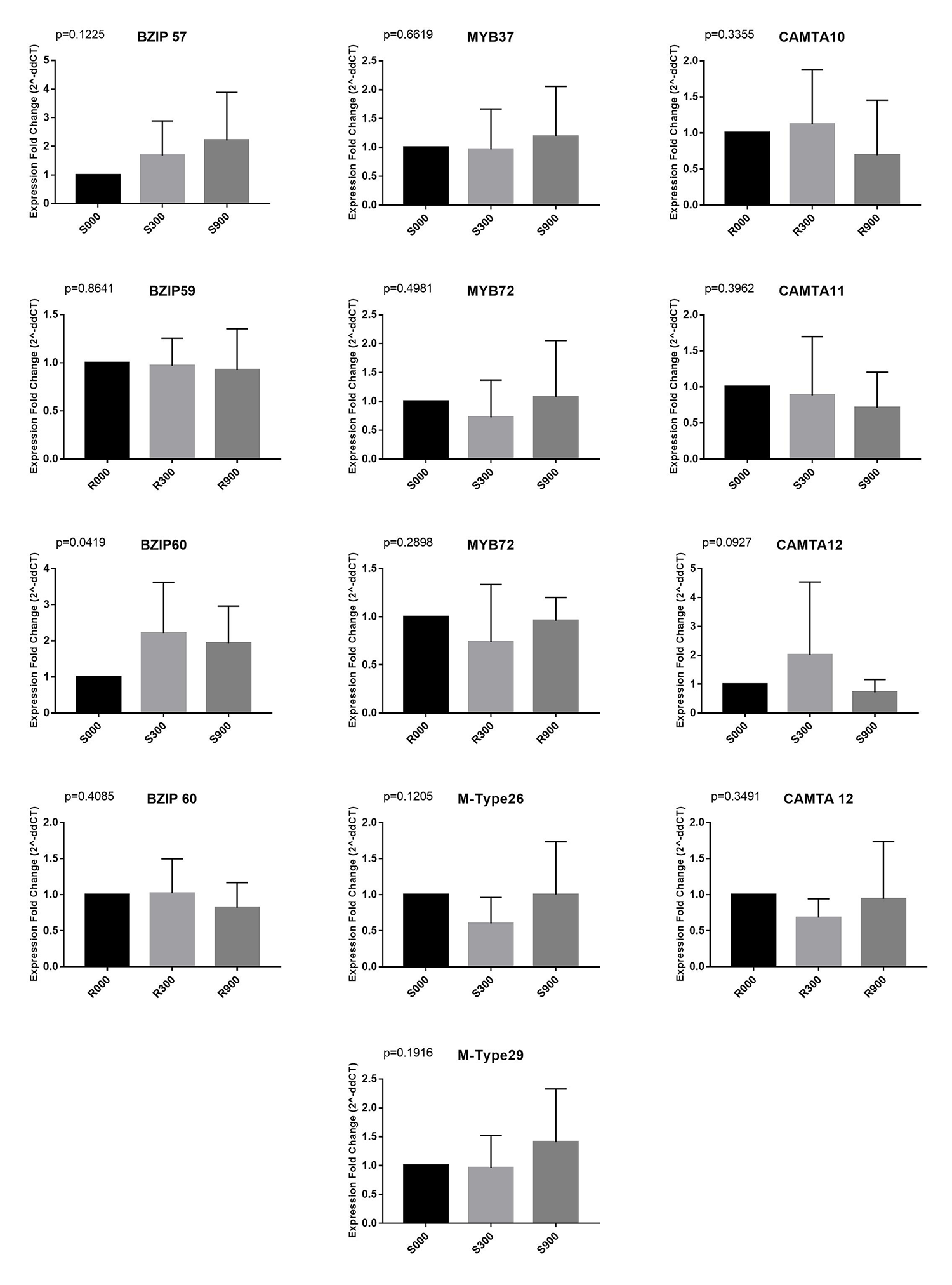

Supplement: S7 Fig — Each graph shows the qRTPCR results for test genes. The annotated putative genes are titled and the mean fold change represented by the 2-ΔΔCT method relative to 0 mM treated samples are shown on the y axis. Error bars depict the standard error of the mean for 3 biological replicates. R000 (roots at 0 mM NaCl), R300 (roots at 300 mM NaCl), R900 (roots at 900 mM NaCl), S000 (shoots at 0 mM NaCl), S300 (shoots at 300 mM NaCl), S900 (shoots at 900 mM NaCl). (TIF) [file pone.0222940.s008.tif]
